# Three-Tiered Risk Stratification Model to Predict Progression in Barrett's Esophagus Using Epigenetic and Clinical Features

**DOI:** 10.1371/journal.pone.0001890

**Published:** 2008-04-02

**Authors:** Fumiaki Sato, Zhe Jin, Karsten Schulmann, Jean Wang, Bruce D. Greenwald, Tetsuo Ito, Takatsugu Kan, James P. Hamilton, Jian Yang, Bogdan Paun, Stefan David, Alexandru Olaru, Yulan Cheng, Yuriko Mori, John M. Abraham, Harris G. Yfantis, Tsung-Teh Wu, Mary B. Fredericksen, Kenneth K. Wang, Marcia Canto, Yvonne Romero, Ziding Feng, Stephen J. Meltzer

**Affiliations:** 1 Division of Gastroenterology and Hepatology, Department of Medicine, Johns Hopkins University School of Medicine, Baltimore, Maryland, United States of America; 2 Division of Gastroenterology and Hepatology, Department of Medicine, University of Maryland School of Medicine, Baltimore, Maryland, United States of America; 3 Medical Department, Ruhr University Bochum, Bochum, Germany; 4 Department of Anatomic Pathology, Baltimore Veterans Affairs Maryland Health Care Systems, Baltimore, Maryland, United States of America; 5 Department of Pathology, Mayo Clinic Foundation, Rochester, Minnesota, United States of America; 6 Division of Gastroenterology and Hepatology, Mayo Clinic Foundation, Rochester, Minnesota, United States of America; 7 Public Health Sciences Division, Fred Hutchinson Cancer Research Center, Seattle, Washington, United States of America; 8 Department of Nanobio Drug Discovery, Graduate School of Pharmaceutical Sciences, Kyoto University, Kyoto, Japan; Deutsches Krebsforschungszentrum, Germany

## Abstract

**Background:**

Barrett's esophagus predisposes to esophageal adenocarcinoma. However, the value of endoscopic surveillance in Barrett's esophagus has been debated because of the low incidence of esophageal adenocarcinoma in Barrett's esophagus. Moreover, high inter-observer and sampling-dependent variation in the histologic staging of dysplasia make clinical risk assessment problematic. In this study, we developed a 3-tiered risk stratification strategy, based on systematically selected epigenetic and clinical parameters, to improve Barrett's esophagus surveillance efficiency.

**Methods and Findings:**

We defined high-grade dysplasia as endpoint of progression, and Barrett's esophagus progressor patients as Barrett's esophagus patients with either no dysplasia or low-grade dysplasia who later developed high-grade dysplasia or esophageal adenocarcinoma. We analyzed 4 epigenetic and 3 clinical parameters in 118 Barrett's esophagus tissues obtained from 35 progressor and 27 non-progressor Barrett's esophagus patients from Baltimore Veterans Affairs Maryland Health Care Systems and Mayo Clinic. Based on 2-year and 4-year prediction models using linear discriminant analysis (area under the receiver-operator characteristic (ROC) curve: 0.8386 and 0.7910, respectively), Barrett's esophagus specimens were stratified into high-risk (HR), intermediate-risk (IR), or low-risk (LR) groups. This 3-tiered stratification method retained both the high specificity of the 2-year model and the high sensitivity of the 4-year model. Progression-free survivals differed significantly among the 3 risk groups, with p = 0.0022 (HR *vs.* IR) and p<0.0001 (HR or IR *vs.* LR). Incremental value analyses demonstrated that the number of methylated genes contributed most influentially to prediction accuracy.

**Conclusions:**

This 3-tiered risk stratification strategy has the potential to exert a profound impact on Barrett's esophagus surveillance accuracy and efficiency.

## Introduction

Barrett's esophagus (BE) is a premalignant condition in which normal squamous epithelium is replaced by a specialized metaplastic, small intestine-like, columnar lining[1]. BE predisposes patients to the future development of esophageal adenocarcinoma (EAC)[1], [2]. The molecular mechanism of the Barrett's esophagus carcinogenic sequence (Barrett's esophagus mucosa, mild and severe dysplasia, to esophageal adenocarcinoma) has not been fully understood. It is believed that long-term inflammation due to gastro-esophageal reflux may cause genetic and epigenetic alterations in Barrett's esophagus, and that accumulation of these genetic and epigenetic alterations would lead the acquisition of malignant characteristics in the Barrett's cells, such as dysregulated cell proliferation, impaired apoptosis, and angiogenesis. As genetic alterations, loss of p16 gene expression (by deletion), the loss of p53 expression (by mutation and deletion), the increase in cyclin expression, and the losses of Rb, APC as well as various chromosomal loci in the Barrett's esophagus have been reported[3]. In addition, promoter hypermethylation of tumor suppressor genes (p16, APC, RUNX3, HPP1, TIMP3, *etc.*) have been observed in the course of Barrett's esophageal carcinogenesis[4].

Because of this increased cancer risk, patients with BE traditionally undergo endoscopic surveillance at regular intervals, usually every two to three years if no additional abnormal findings are present[1], [5]. Therefore, patients often undergo as many as ten or more surveillance endoscopies during a lifetime. In the United States, there are approximately 86.2 million whites between the ages of 45 and 80 years[6]. With a presumed BE prevalence rate of 1.6%[7] for whites, approximately 1.38 million of these subjects have BE. However, because the incidence of EAC in BE is uncommon (approximately 1/200 patient-years), most surveillance endoscopies in BE patients do not detect cancer. Therefore, Barrett's esophagus surveillance would benefit from effective markers to stratify patients according to their level of cancer progression risk.

The currently accepted marker for cancer risk is histologic dysplasia, with high-grade dysplasia (HGD) being considered more accurate than low-grade dysplasia (LGD)[8], [9]. In many centers, confirmed HGD is treated in the same manner as is early-stage EAC, by endoscopic mucosal ablation[10], [11], photodynamic therapy[10], or surgical esophagectomy[12]. In contrast to HGD, the predictive value of LGD for cancer risk assessment is controversial[9], [13]. Moreover, poor reproducibility (high inter-observer variation[8], [14]) in histologic assessment often makes clinical risk assessment problematic. Thus, more accurate tissue-based biomarkers capable of predicting the risk of progression to HGD or EAC would be highly useful.

For the past several years, several groups have studied the role of DNA methylation in esophageal EAC development and progression[4]. Aberrant DNA methylation occurs early in this process, specifically in BE, and methylation increases in frequency in LGD and HGD, becoming most common in EAC[4], [15]. We have shown that certain tumor suppressor genes that undergo methylation in BE can function as biomarkers, predicting whether BE patients will or will not develop HGD or EAC[15].

In actual clinical circumstances, it is difficult to develop prediction models exhibiting both high sensitivity and specificity. When the cutoff point of a prediction outcome is selected to maximize sensitivity, specificity will suffer, and false positives will increase. Conversely, if the cutoff point of a prediction outcome is chosen for high specificity, sensitivity will be lower. To solve this dilemma, we propose a 3-tiered stratification approach. With this method, patients are stratified into either high-risk (HR), intermediate-risk (IR), or low-risk (LR) groups. In the current manuscript, we demonstrate the prediction accuracy, statistical significance, and potential clinical impact of this three-tiered risk stratification system.

## Materials and Methods

### HGD as an outcome endpoint

HGD and EAC are not the same biological or clinical entity. Thus, combining them into a single neoplastic progression endpoint may appear nonstringent[15]. However, at the level of clinical utility, a pronounced shift in management strategy (*i.e.,* more intensive endoscopic surveillance and/or therapeutic intervention) occurs when HGD is diagnosed in BE[10]. For this reason, the progression endpoint was defined as either HGD or EAC.

### Definition of Barrett's esophagus progressor patients and specimens

Previously[15], we defined BE progressor *patients* as BE subjects with either no dysplasia or LGD who later developed HGD or EAC, while progressor *specimens* were defined as any BE *tissues* obtained prior to the progression endpoint. However, in the clinical setting, it is important to know whether or not BE will progress prior to the next scheduled endoscopy. For this reason, in the current study, *progressor specimens* (P) were divided into 3 subgroups: P(0-2), P(2-4), and P(4-), defined as BE or LGD tissues obtained at 0–2 years, 2–4 years, or more than 4 years prior to the progression endpoint, respectively. Similarly, *non-progressor specimens* (NP) were defined as BE or LGD tissues obtained at 0–2 years [NP(0-2)], 2–4 years [NP(2-4)], or more than 4 years [P(4-)] before the non-progression follow-up date.

### Patients and Tissues

Patients undergoing endoscopy at the University of Maryland Medical Center, the Baltimore VA Hospital, and the Mayo Clinic provided written informed consent under a protocol approved by the Institutional Review Boards at each respective institution. Biopsies were taken using a standardized protocol. At each endoscopy, four-quadrant biopsies were obtained at 2-cm intervals throughout the grossly apparent BE segment (or at 1-cm intervals on follow-up after an endoscopy with LGD). Research tissues were obtained from aliquots of grossly apparent Barrett's epithelium. Simultaneously obtained parallel aliquots were sent for histological examination. Diagnoses of BE and dysplasia were made by two experienced gastrointestinal pathologists at the two participating institutions (T-TW and HGY).

**Figure 1:**
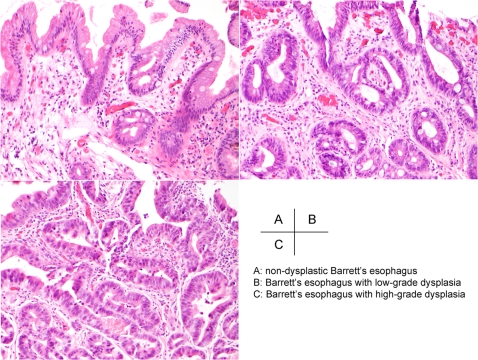
H&E staining of biopsy specimens from patients with Barrett's esophagus. Objective criteria that were used to distinguish LGD and HGD have been published previously[14].

We used an objective criteria for distinguishing LGD and HGD that has been published previously[14] (**Figure 1**). A total of 118 tissue specimens derived from 62 patients with BE constituted the subjects of this study (**Table 1**).

**Table 1:** Numbers of *Tissue Samples and Patients,* Classifications, and Sources.

A) Numbers of Tissue Samples.					
	P(0-2)	P(2-4)	P(4-)	NP	total
Institute					
MAYO	6	11	14	0	31
UMD	11	7	3	66	87
Pathology					
BE	10	13	13	64	100
LGD	7	5	4	2	18
total	17	18	17	66	118

B) Numbers of Patients.					
	non-Progressor (n = 34)		Progressor (n = 28)		
Pathology	BE	LGD	HGD	EAC	Total
Institute					
MAYO	0	0	12 (5)*	7	19
UMD	27	7	5 (1)*	4	43
total	27	7	17 (6)*	11	62

*P(0-2), P(2-4), and P(4-):* progressor samples obtained 0–2 years, 2–4 years, or more than 4 years before the progression date, respectively; *NP:* non-progressor samples; *MAYO:* Mayo Clinic Foundation; *UMD:* University of Maryland and VA Baltimore Medical Centers; *S.D.:* standard deviation; *BE:* non-dysplastic Barrett's esophagus; *LGD:* low-grade dysplasia, *HGD:* high-grade dysplasia; *EAC:* esophageal adenocarcinoma; *Pathology:* for non-progressor patients, the most neoplastically advanced pathology; for progressor patients, pathology at the study endpoint; ^*^: number of patients developing EAC subsequent to a diagnosis of HGD.

### Protocols for DNA extraction, bisulfite treatment and quantitative methylation-specific PCR (MSP)

Tissue specimens were snap-frozen immediately following biopsy or surgical removal and stored in liquid nitrogen until further processing. Genomic DNA from clinical specimens was extracted using a DNeasy kit (Qiagen, Valencia, CA). DNA was treated with bisulfite to convert unmethylated cytosines to uracils prior to MSP, as described previously[16], [17]. DNA methylation status and levels of three genes (*p16, HPP1,* and *RUNX3*) were determined by real-time quantitative MSP using an ABI 7700 Sequence Detection (Taqman) System, as described previously[16], [17]. Primers and probes for quantitative MSP were as described for *p16* [18], *HPP1* [16], *ACTB* [18], and *RUNX3* [15]. A normalized methylation value (NMV) reflecting the percentage of DNA methylated for the gene of interest (GoI) was defined as follows: NMV = 100×(GoI-S/GoI-FM)/(ACTB-S/ACTB-FM), where GoI-S and GoI-FM represent GoI methylation levels in the specimen and fully methylated DNAs, respectively, while ACTB-S and ACTB-FM correspond to β-actin in the specimen and fully methylated (FM) DNAs, respectively.

### Database construction

The database contained 3 clinical parameters (patient's sex, BE segment length (SL), and pathologic assessment: purely metaplastic BE/BE with indefinite dysplasia *vs.* LGD), and 4 methylation-related parameters (normalized methylation values for *p16* [18], *HPP1* [16], and *RUNX3* [15] and methylation index (MI)). Whether 0, 1, 2, or all 3 of these genes were methylated was scored numerically as the methylation index (M.I.). The methylation status of each gene in each tissue was dichotomized into negative or positive categories, according to an optimal NMV cutoff level determined by ROC curve analysis. Methylation status cutoff points for the 2-year prediction model [P(0-2) vs. P(2-4), P(4-), and NP] were 23.4%, 4.4%, and 2.2% for HPP1, p16, and RUNX3, respectively. Methylation status cutoffs for the 4-year prediction model [P(0-2) and P(2-4) vs. P(4-) and NP] were 16%, 1.12%, and 0.17% for HPP1, p16, and RUNX3, respectively. Cutoffs of the NMV for the 4-year prediction model were lower than those for the 2-year model, possibly because epigenetic alterations were less widespread in progressor tissues at 4 years than at 2 years prior to progression. Thus, 4 clinical features and 4 gene methylation parameters were used to generate prediction models.

### Establishment of prediction models for BE progression

**Figure 2:**
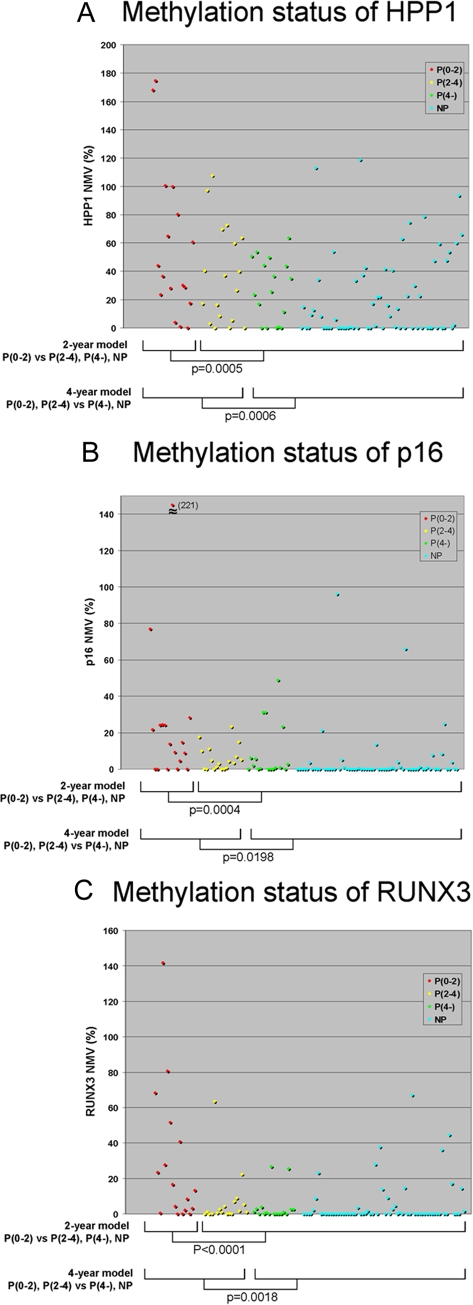
Methylation status of HPP1, p16, and RUNX3. Normalized methylation values (NMVs) of HPP1 (1A), p16 (1B), and RUNX3 (1C) are shown. p: p-value of t-test. NMVs of genes in progression-positive cases [P(0-2) and P(0-2)+P(2-4) for the 2-year and 4-year models, respectively] were significantly higher than NMVs of progression-negative (P(2-4)+P(4-)+NP and P(4-)+NP for the 2-year and 4-year models, respectively).

To stratify patients into 3 groups, *viz.,* high-risk (HR), intermediate-risk (IR), or low-risk (LR), we established two prediction models using linear discriminant analysis (LDA). To select the HR group in the 2-year prediction model, only P(0-2) specimens were defined as progressors for LDA, while all other specimens were defined as nonprogressors. To obtain a prediction value for each specimen, leave-one-out crossvalidation (LOOCV) was performed. Prediction model accuracy was assessed by measuring the area under the ROC curve (AUROC). These models generated prediction output values ranging from 0 to 1, representing highest to lowest risk, respectively. Cutoff points of prediction model outputs defining the HR group were chosen for 90% specificity in order to minimize the number of unnecessary endoscopies (**Figure 2A**). To select the LR group in the 4-year prediction model, both P(0-2) and P(2-4) specimens were defined as progressors for LDA, while other specimens were defined as nonprogressors. Cutoff points of prediction model outputs defining the LR group were chosen to achieve 90% sensitivity, in order to minimize failure in detecting progressor patients (**Figure 2B**). The IR group was defined as specimens belonging to neither the HR nor the LR groups.

When constructing prediction models using multiple parameters, it is important to choose the most optimal parameter set[19]. In the current study, the most optimal parameter set was defined as that possessing the highest AUROC value among 127 ( = 2^7^−1) possible combinations of the 4 epigenetic and 3 clinical parameters.

### Additional statistics

Detailed methods of permutation analysis and incremental value analysis are described in **Text S1**. The progression-free survival of patients in each risk category was analyzed using the Kaplan-Meier method and log-rank testing for statistical significance of differences in progression-free survival. A p-value of less than 0.05 was considered significant. All LDA, LOOCV, and AUROC calculations were performed using Matlab, v.7.0 (Mathworks, Natick, MA). The remaining statistical calculations were performed using STATISTICA v.6.1 (Statsoft, Tulsa, OK).

## Results

### Association between epigenetic parameters and BE neoplastic progression

The NMV of HPP1, p16, and RUNX3 in each specimen is plotted in **Figures 2A, 2B, and 2C**, respectively. For the 2-year prediction model, only P(0-2) specimens were defined as positive for progression, while others were classified as progression-negative for LDA. T-testing demonstrated that the NMVs of all 3 genes in group P(0-2) were significantly higher than their corresponding NMVs in groups P(2-4), P(4-), and NP (p = 0.0005, 0.0004, <0.0001 for HPP1, p16, and RUNX3, respectively). For the 4-year prediction model, P(0-2) and P(2-4) specimens were both defined as positive for progression, while P(4-) and NP specimens were classified as progression-negative for LDA. NMVs of all 3 genes in groups P(0-2) ann P(2-4) were significantly higher than their NMVs in groups P(4-) and NP (p = 0.0006, 0.0198, and 0.0018 for HPP1, p16, and RUNX3, respectively). The discriminant formula for 2- and 4-year prediction models are described in **Text S1**.

In addition, the relationship between MI and BE progression is displayed in **Table 2**. the MI of progression-positive (*) and -negative (^§^) specimens for both the 2-year and 4-year predictions differed significantly by Chi-square testing (p<0.00001).

**Table 2:** Methylation Index (MI) and Barrett's progression.

A. 2-year prediction model					
MI	Sample group				Total
	*P(0-2)	^§^P(2-4)	^§^P(4-)	^§^NP	
0	1	3	4	37	45
1	4	9	7	18	38
2	2	2	4	8	16
3	10	4	2	3	19
Total	17	18	17	66	118

B. 4-year prediction model					
MI	Sample group				Total
	*P(0-2)	*P(2-4)	^§^P(4-)	^§^NP	
0	1	2	4	34	41
1	3	4	3	16	26
2	3	5	7	12	27
3	10	7	3	4	24
Total	17	18	17	66	118

Methylation index of 118 samples were shown. The dichotomization cutoff point of NMV for each gene (methylated vs. unmethylated) was different between 2-year and 4-year model. Therefore, the MI in some samples were different between 2-year and 4-year model. MI of positive (^*^) and negative (^§^) cases were significantly different by chi-square test (for both tables, p<0.00001)

### A combined prediction model of BE neoplastic progression

**Figure 3:**
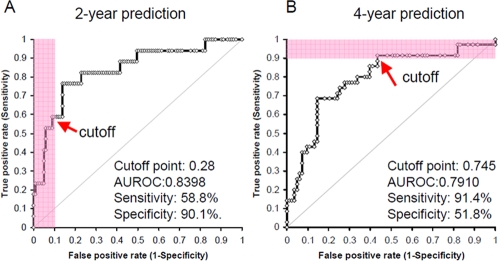
Best ROC curves of 2- and 4-year prediction models. A: For the 2-year prediction model, the best AUROC (0.8387) was obtained using 4 parameters: SL, pathology, p16, and methylation index (MI). Based on this ROC curve, we chose an output value cutoff point defining the HR group to maximize specificity (>90%, *red area*) rather than sensitivity. B: For the 4-year model, the best AUROC (0.7910) was achieved using 3 parameters: SL, pathology, and MI. Based on this ROC curve, we selected an output value cutoff point defining the LR group to maximize sensitivity (>90%, *red area*) rather than specificity.

**Figure 3A** demonstrates the best ROC curve for 2-year prediction, based on the 4 parameters of SL, pathology status, p16 and MI. The AUROC, specificity, and sensitivity of this model were 0.8387 (95% confidence interval (C.I.): 0.7273–0.9501), 90.1%, and 58.8%, respectively. **Figure 3B** displays the best ROC curve for 4-year prediction using the 3 parameters of SL, pathology, and MI. The AUROC, specificity, and sensitivity of the 4-year model were 0.7910 (95% C.I.: 0.6968–0.8853), 91.4%, and 51.8%, respectively. On ROC curves, prediction output values for the 2-year (0.28) and 4-year (0.745) prediction models attained 90% specificity and 90% sensitivity, respectively, therefore these output values were selected as cutoffs to define risk levels (HR or LR; see above).

**Figure 4:**
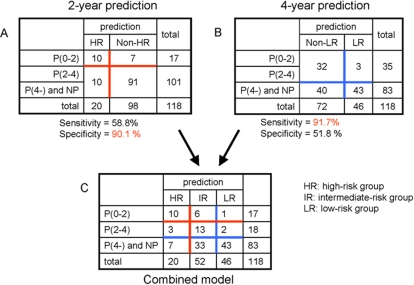
Combining the 2-year and 4-year prediction models. A and B: 2×2 contingency tables for the 2-year and 4-year prediction models, respectively. Cutoff points for the 2-year and 4-year model output values were chosen to attain 90% specificity and sensitivity, respectively, as described above (Figure 3). C: combined 3×3 contingency table. Red and blue cross-lines correspond to red and blue lines in A and B. P(0-2), P(2-4), P(4-): specimens obtained from progressor patients < = 2 years, 2–4 years, or >4 years prior to progression, respectively. NP: specimens derived from non-progressor patients with more than a 4-year follow-up period.

Next, to unify this algorithm, a 3×3 contingency table was generated from two 2×2 contingency tables for the 2-year and 4-year prediction models (**Figure 4**). Among 118 specimens, 20, 52, and 46 specimens were stratified into HR, IR, and LR groups, respectively. Theoretically, specimens could have met both the HR (<0.28 for 2-year model) and the LR (>0.745 for 4-year model) criteria simultaneously. However, in actuality, such an internally contradictory specimen did not occur in the current study. Based on the combined prediction model, this 3-tiered stratification procedure could save more than 5300 endoscopes per year in the United States (**Figure S1)**. In addition, the permutation procedure suggested that our observed results were unlikely to have occurred by chance (**Figure S2**).

### Progression-free survival in the three risk tiers

**Figure 5:**
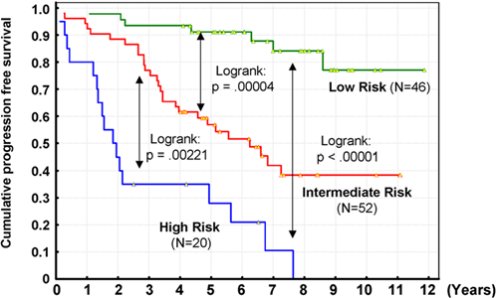
Progression-free survival in the 3 risk tiers. Kaplan-Meier survival curves for each of the 3 risk tiers are shown. The 2-year progression-free survival rates of HR, IR, and LR were 45%, 88.5%, and 97.8%, respectively. Four-year progression-free survival rates were 35%, 63.5%, and 93.5%, respectively. Differences in progression-free survival among these 3 risk tiers were statistically significant (log-rank test).

Three Kaplan-Meier curves showed a statistically significant difference in progression-free survival among the three risk tiers defined by the combined LDA model (**Figure 5** **)**. The LR group had the best progression-free survival, significantly better than both the IR and HR groups (p<0.0001, logrank test). The HR group had the worst progression-free survival, significantly shorter than both the IR (p = 0.0022, logrank test) and LR groups. The IR group had a progression risk significantly different from the other 2 groups. Thus, these 3 specimen groups classified by the combined model assigned progression risk in a meaningful manner.

### Time-course analysis of risk prediction in each patient

**Figure 6:**
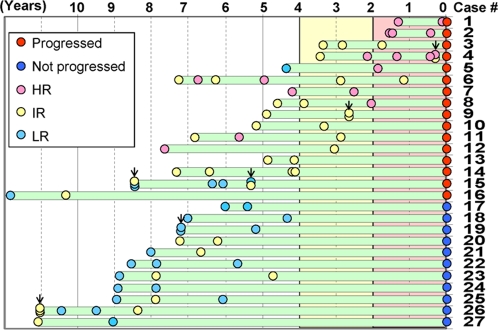
Changes in 3-risk-group prediction during follow-up. The *horizontal axis* represents the time interval preceding the date of progression or nonprogression. Among 62 patients, 27 patients underwent biopsies at multiple timepoints before progression. Each *green bar* represents the follow-up period of each patient, and each *circle* shows the timing of biopsies and the risk level prediction (designated by each circle's color). Vertically overlapped circles *with arrows* indicate multiple tissue specimens obtained at a single endoscopy, while horizontally overlapped circles (no arrows) indicate specimens obtained at temporally neighboring but separate endoscopies.

Criteria of a good biomarker require not only its prediction of outcomes to be highly accurate in cross-sectional studies, but also its changes in value to reflect clinical disease course in longitudinal studies. Therefore, we performed a time-course analysis. In progressor patients, progression risk should increase or be high at least in the short time before the progression, whereas progression risk in non-progressor cases should not increase over time. **Figure 6** demonstrates longitudinal change in risk according to this prediction model in patients who contributed multiple tissue specimens. In actuality, among 16 progressor patients (case #1–16), five HR specimens from 4 patients (cases #4, 6, 11, and 12) were reduced to IR during their follow-up BE surveillance period. However, all 4 cases progressed to HGD at the end of their follow-up period. This finding suggests that even if a BE patient previously diagnosed as HR is reduced to IR at a follow-up endoscopic biopsy, this BE patient should be followed at the HR time interval (*i.e.,* once yearly), rather than at the IR interval (once every 2 years). In addition, there was not a single patient whose risk assessment was reduced from HR to LR. In contrast, risk assessments for all 11 non-progressor patients (cases #17–27) stayed in LR or IR, while no non-progressor patient's risk assessment increased to HR.

In patients with marginal risk levels, risk assessment sometimes fluctuated between LR and IR. Specifically, “Upgrading” of risk from LR to IR occurred in 4 non-progressor patients, as well as in 2 progressor patients more than 5 years before progression. Conversely, risk “downgrading” from IR to LR was observed in 3 non-progressor patients, as well as in one progressor patient (case #15) more than 6 years prior to progression.

### Incremental value analysis

In **Table 3** **,** differences between AUROCs in parameter sets with (plus) *vs.* without (minus) a given parameter represent the portion contributed to prediction accuracy of each parameter (*i.e.,* its incremental value). In both the 2-year and 4-year prediction models, methylation index (MI) exerted the greatest impact on prediction accuracy (0.0977 and 0.0857 in the 2-year and 4-year prediction models, respectively), while pathology (non-dysplastic BE *vs.* LGD) was the second-most influential parameter (0.0542 and 0.0462 in the 2-year and 4-year prediction models, respectively).

**Table 3:** Univariate analyses of incremental values.

	sex	SL	pathology	HPP1	p16	Runx3	MI
2-year prediction		*	*		*		*
minus	0.7546	0.7232	0.7178	0.7335	0.7211	0.7276	0.6932
plus	0.7402	0.7625	0.7720	0.7518	0.7644	0.7579	0.7908
increment	−0.0144	0.0393	0.0542	0.0183	0.0433	0.0303	0.0977
p-value	<0.00001	0.00048	<0.00001	0.13229	0.00058	0.01643	<0.00001
4-year prediction		*	*				*
minus	0.7057	0.6882	0.6879	0.6944	0.6919	0.6981	0.6652
plus	0.7192	0.7298	0.7341	0.7224	0.7254	0.7198	0.7509
increment	0.0135	0.0416	0.0462	0.0280	0.0335	0.0216	0.0857
p-value	<0.00001	0.00012	<0.00001	0.01876	0.00576	0.02582	<0.00001

*SL*: segment length; *MI*: methylation index; *minus*: median values of AUROCs in variable sets lacking indicated parameter; *plus*: median values of AUROCs in variable sets containing indicated parameter; *increment*: differences of median values in minus and plus, which represents the impacts of individual parameters; ^*^: parameters selected in the best parameter sets; p-value: p-values of paired t-tests.

## Discussion

Compared to the general population, BE patients have a 30-125-fold increased risk of developing EAC [20]. Therefore, periodic endoscopic surveillance is generally practiced in the management of BE patients[21]. EAC detected during BE surveillance tends to occur at an earlier stage and have a better prognosis than EAC found in the non-surveillance setting[22], [23]. However, in terms of cost-effectiveness, the impact of current BE surveillance recommendations is controversial [24], [25], because the progression rate of BE to EAC is very low. Thus, stratification of BE patients to improve BE surveillance efficiency would be beneficial in terms of cost-effectiveness, as well as represent an improvement in quality of life due to diminished anxiety and inconvenience.

Current recommendations for the appropriate BE follow-up interval are as follows: two initial annual endoscopies, followed by a 3-year interval for BE cases without dysplasia, or less than 1 year for BE with LGD until dysplasia is no longer found[21]. To simplify calculations in the current study, we compared endoscopy savings between a uniform 2-year follow-up protocol and our three-tiered model. Using a simulation, we estimated that this 3-tiered risk stratification strategy would save approximately 5,300 endoscopies annually in the United States. If a 0.13% overall upper GI endoscopy complication rate is assumed, this endoscopy savings would prevent 6.9 unnecessary complications annually in the United States[26].

These three risk tiers were defined using only progression status at 2 years and 4 years after analyzed specimens were obtained. Thus, theoretically, this stratification cannot guarantee differences in progression-free survival more than 4 years after sampling. However, Kaplan-Meier progression-free survival analysis (**Figure 5**) demonstrated that our prediction model could discriminate among the 3 risk groups well not only at 2 and 4 years post-sampling, but also over the entire follow-up period.

The Kaplan-Meier progression free survival curve showed that some LR patients progressed soon after the fourth year following their initial (index) BE EGD. However, the model recommends follow-up endoscopy within 4 years after any LR EGD. For example, patient #5 in **Figure 6** had a LR specimen at 4.5 years prior to progression. This case does not represent a flaw in the model, since followup EGD was indeed performed as per the model's recommendation, and his risk level at 1.8 years before progression was upgraded to HR.

In this study, there were 6 sets of multiple specimens from the same timepoint in 5 patients (patients 4, 9, 15, 19, and 26; indicated by *arrows* in **Figure 6**). The trained prediction model yielded conflicting risk grade outputs in 3 specimen sets (patients 4, 15, and 26). One possible explanation for this observed discrepancy was variation in biopsy sampling. Carcinogenic events, including histologic[27], genomic[28], and epigenetic alterations[18], do not occur uniformly throughout the BE epithelium. Therefore, as with histologic assessment, this discrepancy could have been caused by biopsy sampling variation. One potential solution to this issue is to perform sampling from multiple anatomic loci, as in histological assessment during current BE surveillance, and to apply the highest risk assessment obtained from these multiple loci to scheduling of the next BE surveilance endoscopy.

Our incremental analysis demonstrated that MI made a much greater contribution to prediction accuracy than did the other parameters. These findings are not surprising, since some researchers have reported that MI or CpG Island Methylator Phenotype (CIMP) status correlates with patient survival in esophageal cancer[29] and other malignancies, such as colorectal cancer[30] or neuroblastoma[31]. However, mechanism(s) by which an “MI-high” epigenetic or methylator phenotype contributes to carcinogenesis remain(s) unclear. Possible explanations include: 1) methylator phenotype-positive tumors tend to be hypermethylated in promoter regions of other genes, including tumor suppressor genes (such as APC, CDH1, TIMP3, and others) [4]; 2) methylator phenotype-positive tumors tend to undergo hMLH1 gene inactivation via promoter hypermethylation. Although hMLH1 hypermethylation is relatively uncommon in EAC compared to gastric, colorectal, or endometrial cancer[32], hMLH1 hypermethylation in BE may cause microsatellite instability in the coding regions of the tumor suppressor genes[33]; 3) a methylator phenotype may be associated with chromatin remodeling[34]; and 4) methylated cytosines are hotspots for mutations, as with the p53 gene[35].

Histopathologic assessment of dysplasia in BE is currently the most widely accepted parameter with which to predict BE progression. However, histopathologic assessment is plagued by inter-observer variation, which can lead to confusion during clinical BE surveillance. One aim in this study was to develop biomarkers that were more objective and quantifiable than histopathologic assessment, such as epigenetic parameters (including MI). However, MI data also risk being influenced by several factors. One such factor is the dichotomization of normalized methylation values (NMV) for each gene into positive *vs.* negative classes. The significance and relevance to BE progression of methylation of each gene may vary. For this reason, we did not use uniform criteria to dichotomize NMV data, but rathere optimized criteria for each gene based on ROC curve analysis. Another factor potentially influencing MI data is endoscopic sampling bias. Methylation status in BE occurs heterogeneously [32], as does genomic clonality [28]. Therefore, multiple biopsies during each endoscopic procedure are widely in BE surveillance.

In both the 2-year and 4-year prediction models, according to incremental value analysis, pathological assessment was the second-most influential parameter. The natural history of LGD is not well-described, with ultimate progression to HGD and EAC ranging from 5-12.5% [36], [37]. LGD also frequently regresses to BE, at rates ranging as high as 60-75% [38], [39]. In addition, the histological diagnosis of dysplasia in BE[14], [40], as well as in other premalignant lesions (esophageal squamous epithelium[41], stomach[42], ulcerative colitis[43], and others), is characterized by high inter-observer variability. Therefore, the value of LGD as a clinical cancer risk marker is controversial. However, some studies have demonstrated that LGD is a risk factor for the development of EAC in BE [13], [39]. Our findings corroborated this predictive value of LGD. In addition, our results emphasize the power of combining pathological assessment with methylation status to improve risk prediction accuracy.

In both the 2-year and 4-year models, segment length (SL) was also one of the parameters in the most optimal parameter set. Patients with long-segment (≥3cm) BE are widely believed to carry a greater risk of developing EAC than those with short-segment BE [20]. However, other studies have demonstrated that the risk of developing EAC in patients with short-segment BE is not substantially lower than in patients with long-segment BE[44]. In the current study, SL was selected in the optimal parameter set for both the 2-year and the 4-year models (**Table 2**). This finding also suggests that SL is not strong as an independent clinical marker; however, it does contribute significantly as a member of a parameter set.

The NCI Early Detection Research Network (EDRN) defined five phases of biomarker development in the early detection of cancer[45]. Currently, flow cytometric (tetraploidy, aneuploidy)[46] and loss of heterozygosity (LOH) at the p53 locus[47] have advanced regarding biomarker validation in large-scale phase 4 studies as defined by EDRN classification. However, the AUROC for prediction of BE progression (to EAC) based on flow cytometry was 0.76[46]. Thus, our multi-tiered prediction method based on clinical and epigenetic parameters (AUROC = 0.8387 and 0.7910 for the 2-year and 4-year models, respectively) exceeded published AUROCs based on single flow cytometric analysis alone[46]. Moreover, assessment of aberrant methylation in BE can be performed using formalin-fixed, paraffin-embedded specimens[48]. Finally, matching normal tissue is not necessary for methylation assays, in contrast to LOH. These advantageous features of methylation-based biomarkers may make specimen collection easier, thereby facilitating large-scale multi-institutional prospective or retrospective studies.

The work described in this report is now the subject of an EDRN Phase 3 validation study. In preparing to proceed to Phase 4 validation, we developed a prediction model to stratify BE patients, validated our model, and estimated its potential clinical impact (endoscopy savings) by applying a simulation. Because of the rarity of BE progressor specimens, the number of progressor patients and specimens was relatively small, despite collecting them from two institutions. Further studies may be needed to increase the number of BE progressor and non-progressor specimens by collecting specimens from multiple additional institutions prior to initiating a prospective Phase 4 study.

In conclusion, we developed a 3-tiered risk stratification strategy for neoplastic progression prediction in BE patients, based on epigenetic and clinical parameters. This strategy offers considerable promise to benefit the current BE surveillance health care system.

## Supporting Information

Figure S1 Simulation of endoscopy savings. Sample (P vs. NP) proportions in this study are not identical to those in the clinical setting. Thus, to estimate real sample proportions, we converted Figure 4C, based on assumptions that the progression rate of BE in 4 years ( = (P(0-2)+P(2-4))/(P(4-)+NP)) would be 1/25, and that the proportion of HR, IR and LR in each sample group (P(0-2), P(2-4), P(4-),NP) would be identical to proportions observed in Figure 4C. In addition, total BE patient number was adjusted to the estimated number (68,932) of currently diagnosed BE patients in the United States. Numbers of endoscopies needed were calculated for a 2-year uniform follow-up protocol and for our three-tiered stratification approach.
(0.74 MB TIF)

Figure S2 Permutation analysis. The arrow indicates the AUROCs (0.8387 and 0.7910) of the original 2-year and 4-year prediction models, respectively. Among 1000 AUROCs generated by the permutation analysis, 3 and 4 AUROCs surpassed the original AUROCs for 2- and 4-year model, respectively. This permutation analysis indicated that AUROC in our original prediction model were significantly better than AUROCs of the null hypothesis, with a false discovery rate (FDR) of 0.003 and 0.004 for 2- and 4-year model, respectively.
(0.92 MB TIF)

Text S1 1. Formula of Discriminant function 2. Estimation of surveillance endoscopy savings 3. Incremental value analysis 4. Permutation Analysis.
(0.06 MB DOC)
