# Supplementary figures and images for: Three-Tiered Risk Stratification Model to Predict Progression in Barrett's Esophagus Using Epigenetic and Clinical Features
Source: PLoS One. 2008 Apr 2;3(4):e1890. doi: 10.1371/journal.pone.0001890 (PMC2270339; doi:10.1371/journal.pone.0001890)

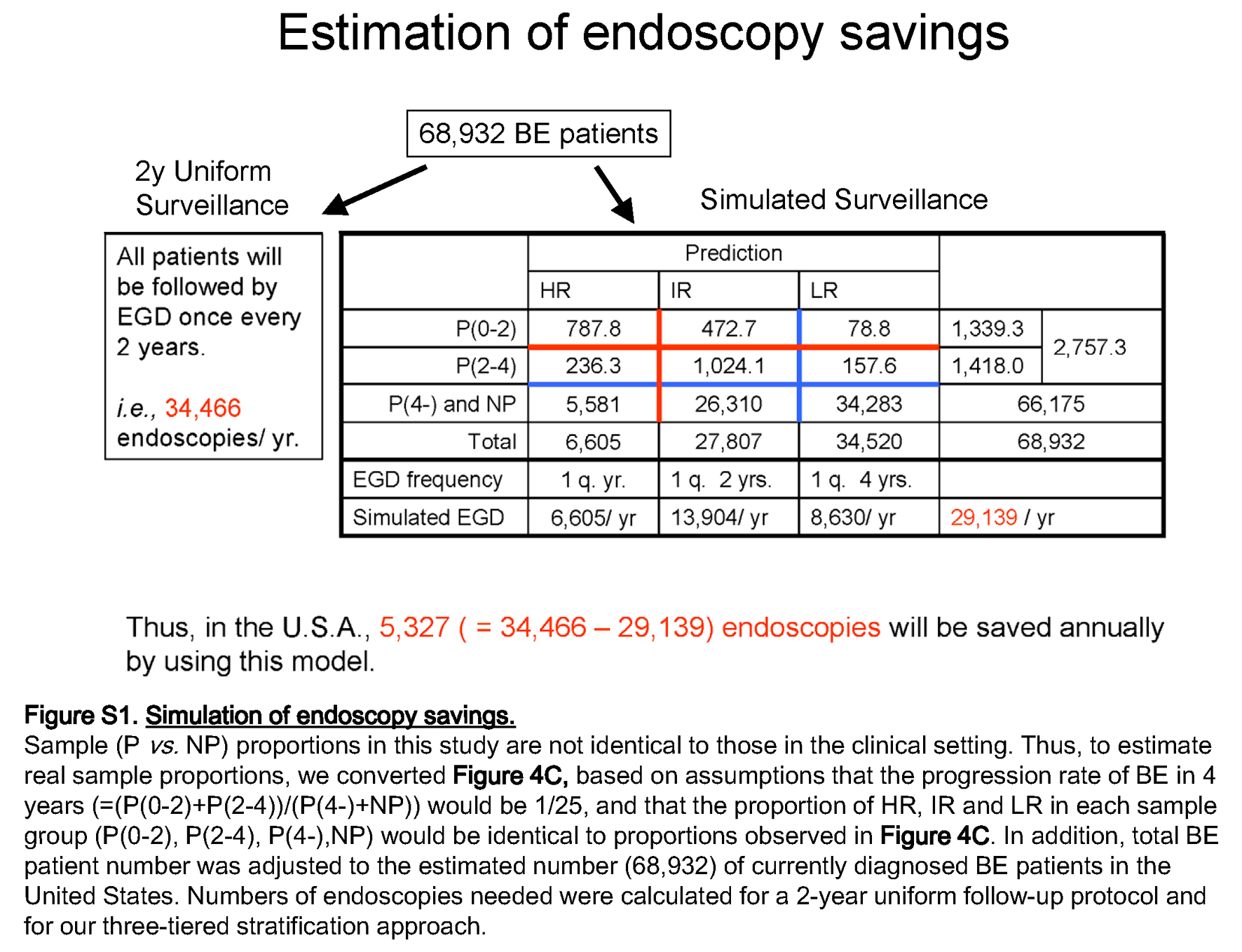

Supplement: Figure S1 — Simulation of endoscopy savings. Sample (P vs. NP) proportions in this study are not identical to those in the clinical setting. Thus, to estimate real sample proportions, we converted Figure 4C, based on assumptions that the progression rate of BE in 4 years ( = (P(0-2)+P(2-4))/(P(4-)+NP)) would be 1/25, and that the proportion of HR, IR and LR in each sample group (P(0-2), P(2-4), P(4-),NP) would be identical to proportions observed in Figure 4C. In addition, total BE patient number was adjusted to the estimated number (68,932) of currently diagnosed BE patients in the United States. Numbers of endoscopies needed were calculated for a 2-year uniform follow-up protocol and for our three-tiered stratification approach. (0.74 MB TIF) [file pone.0001890.s001.tif]

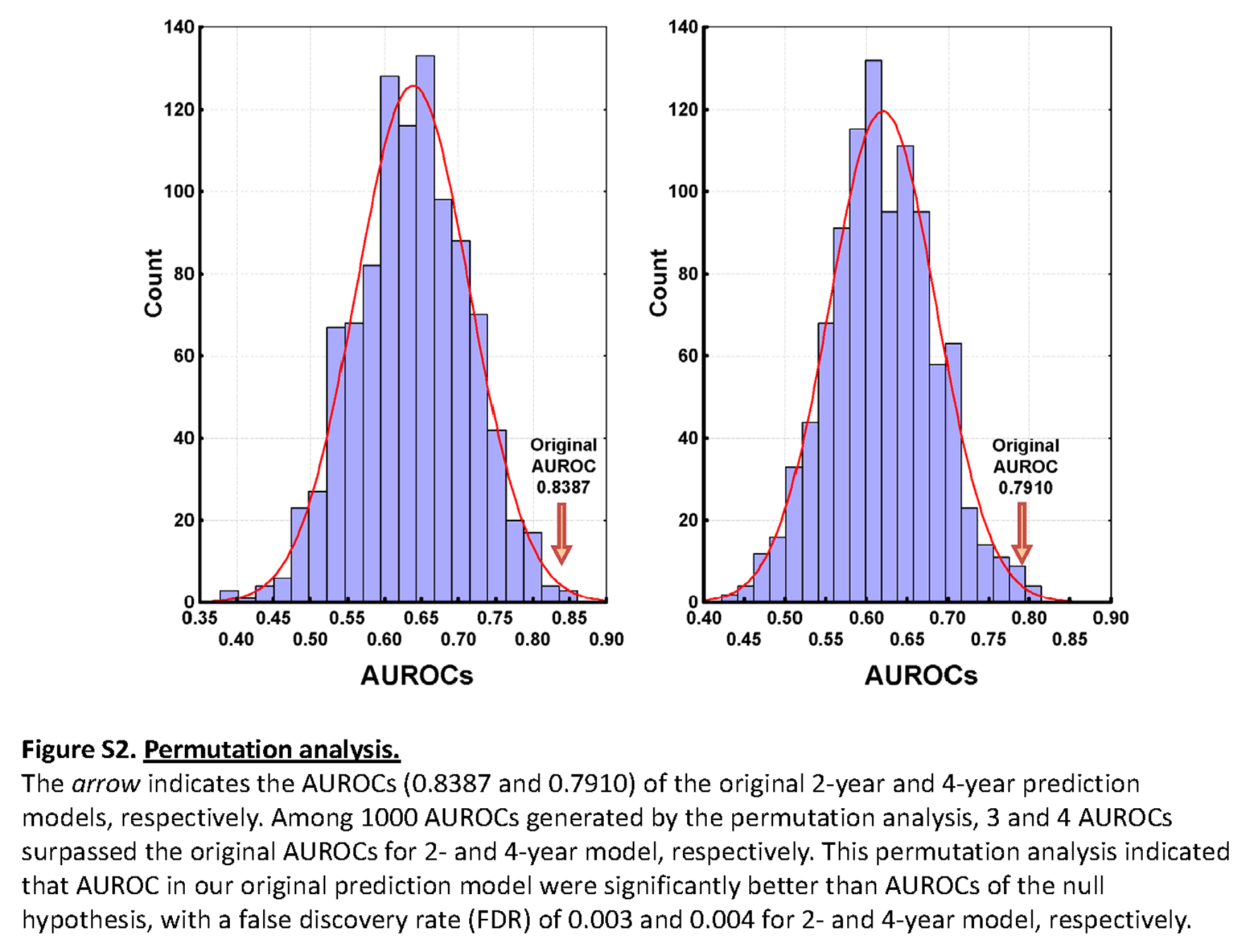

Supplement: Figure S2 — Permutation analysis. The arrow indicates the AUROCs (0.8387 and 0.7910) of the original 2-year and 4-year prediction models, respectively. Among 1000 AUROCs generated by the permutation analysis, 3 and 4 AUROCs surpassed the original AUROCs for 2- and 4-year model, respectively. This permutation analysis indicated that AUROC in our original prediction model were significantly better than AUROCs of the null hypothesis, with a false discovery rate (FDR) of 0.003 and 0.004 for 2- and 4-year model, respectively. (0.92 MB TIF) [file pone.0001890.s002.tif]
