# Supplementary material for: Three-Tiered Risk Stratification Model to Predict Progression in Barrett's Esophagus Using Epigenetic and Clinical Features
Source: PLoS One. 2008 Apr 2;3(4):e1890. doi: 10.1371/journal.pone.0001890 (PMC2270339; doi:10.1371/journal.pone.0001890)
Supplement: Text S1 — 1. Formula of Discriminant function 2. Estimation of surveillance endoscopy savings 3. Incremental value analysis 4. Permutation Analysis. (0.06 MB DOC) [file pone.0001890.s003.doc]

Supplemental text

Three-tiered Risk Stratification Model to Predict Progression in Barrett’s Esophagus Using Epigenetic and Clinical Features

Fumiaki Sato, Zhe Jin, Karsten Schulmann, Jean Wang, Bruce D. Greenwald, Tetsuo Ito, Takatsugu Kan, James P. Hamilton, Jian Yang, Bogdan Paun, Stefan David, Alexandru Olaru, Yulan Cheng, Yuriko Mori, John M. Abraham, Harris G.Yfantis, Tsung-Teh Wu, Mary B. Fredericksen, Kenneth K. Wang, Marcia Canto, Yvonne Romero, Ziding Feng, Stephen J. Meltzer

1. Formula of Discriminant function

Formula of the discriminant function for 2-year model using the optimized data set of all samples (N=118) was

z = - 0.117914343 × (segment length (cm)) - 2.156941579 ×( pathology (0: no dysplasia or indefinite dysplasia, 1: low-grade dysplasia)) - 0.032045818 ×(NMV of p16) -1.182896489 × (methylation index) + 3.960081783.

The cutoff point of the z-value was -0.75 (z<-0.75: high-risk group, z>-0.75: not high risk group).

And, formula for 4-year model was

z = - 0.044624436 × (segment length (cm)) - 2.052983023 ×( pathology (0: no dysplasia or indefinite dysplasia, 1: low-grade dysplasia)) - 1.029305669 × (methylation index) + 2.362119227.

The cutoff point of the z-value was 0.99 (z<0.99: not low-risk group, z>0.99: low-risk group).

2. Estimation of surveillance endoscopy savings

Method and results

The progressor to non-progressor sample ratio in the current study was not identical to that in the actual clinical setting. Thus, expected surveillance endoscopy savings in clinical practice could not be estimated directly from our raw data. To estimate the potential endoscopy savings from our strategy, our raw data table (**Figure 3C**) was first converted into a simulated table (**Figure S1**), based on the estimated prevalence of BE and its progression rate. In the United States, there are currently 86,165,180 whites between the ages of 45 and 80 years[1]. With a presumed BE prevalence rate of 1.6%[2], 1,378,643 of these subjects have BE. Since only 5% of EAC patients carry a known diagnosis of BE prior to developing EAC[3], we estimated that only 5% (68,932) of persons with BE currently have been under BE surveillance. EAC risk in BE patients is approximately 1 per 200 patient-years[4]. However, to our knowledge, no report has directly estimated the combined risk of progression from BE to either HGD or EAC. Several authors have studied the risk of progression from HGD to EAC, varying from 16% to 59%[5,6]. Moreover, unsuspected EAC was found in 0 to 73% (average: 59%=80/140) of specimens resected from BE patients with HGD[7]. Therefore, we assumed that 50% of HGD cases would progress to EAC. Based on an EAC risk in BE patients 1/200 patient-years, 1/50 BE patients would develop EAC during a 4-year follow-up window. And presuming a 50% progression rate from HGD to EAC, 1/25 (2,757.3/68,932) BE patients would develop HGD during a 4-year follow-up period. A schematic illustration of this simulation (**Figure S1**) displays the number of endoscopies estimated to occur in diagnosed BE patients. Under a uniform 2-year follow-up protocol (1/2 times per year), 34,466 endoscopies per year would customarily be performed in these BE subjects. Under our 3-tiered classification model, 6,605 HR cases, 27,807 IR cases, and 34,520 LR cases would be endoscoped 1, 1/2, or 1/4 times per year, respectively; thus, 29,139 total endoscopies per year would be performed on these BE subjects. Therefore, the number of endoscopies would be reduced by 5,327 annually based on this simulation (**Figure S1**). Moreover, our three-tiered stratification system, once validated, could reduce anxiety in 34,441 (50.0%) of BE patients who would now be classified as LR.

Discussion

It should be noted that estimated endoscopy savings were adversely affected by the LR diagnosis rate (the cutoff point of the 4-year model) being greater than the HR diagnosis rate (the cutoff point of the 2-year model). If a less conservative cutoff had been chosen, endoscopy savings would have been higher. However, the endoscopy savings rate depends on our tolerance of false-negative prediction. We believe that the potential benefits of this strategy (15.5% endoscopy savings, reduction of surveillance interval in 50.0% of BE patients, and earlier detection of EAC in the HR group) outweigh its potential drawback of a 5.9% (78.8/1339.3, **Figure 4**) false-negative prediction rate.

BE prevalence was estimated for the United States white population. BE prevalence in African-American and Asian populations is much lower than in whites. However, the Hispanic population has a prevalence of BE similar to that of whites [8]. A study of the progression rate of BE and BE surveillance efficacy in the Hispanic population *per se* has not been published. However, our estimations of BE prevalence and surveillance endoscopy may have been overly conservative. Thus, our strategy has the potential to save more endoscopies per year than we estimated.

Markov modeling is used to estimate the economic impact of BE surveillance[9]. However, showing dollar amounts saved by reducing endoscopies might have been misleading, because many other costs must also be considered. For example, money-saving factors also include the value of fewer endoscopy complications, earlier diagnoses (endoscopic therapy *vs.* surgical intervention), and fewer cancer deaths (terminal care). Conversely, money-consuming factors include costs of methylation assays and unnecessary medical care for false-negative cases. Moreover, there are many uncertain factors, such as the costs of methylation assays (not yet commercially available) and prediction accuracy (not yet tested in a prospective study). Therefore, in the current manuscript, we restricted our analysis to the benefits of this strategy in the terms of clinical outcome, rather than economic impact.

3. Incremental value analysis

Method

The concept of incremental value analysis resembles that of biologic loss-of-function analysis. In biologic loss-of-function studies, for example, researchers knock one gene out, observe phenotypic changes, and deduce the function of the gene knocked out. In the current incremental value analysis, we eliminated one or multiple parameters from the dataset, then compared prediction accuracy before *vs.* after elimination of the parameter. To assess incremental contributions of individual epigenetic or clinical parameters toward prediction accuracy, we compared AUROCs of models lacking *vs.* containing each parameter. The significances of differences between AUROCs were determined by paired t-testing. For example, to assess the incremental value of parameter A, we divided AUROC values into 2 groups (AUROCs with *vs*. without A), such as parameter B alone *vs*. parameters A+B, parameter C alone *vs*. parameters A+C, parameters B+C *vs*. parameters A+B+C, etc. In total, we performed paired t-testing for 2 groups of AUROC values consisting of 63 (= 2^6^-1) pairs of AUROC values by generating all possible pairs of variable sets. This large AUROC variation set comprised a paired analysis of all the variable sets derived by adding or subtracting each individual methylation or clinical parameter. For the incremental value of a given group of parameters, the best AUROCs of variable sets with and without the particular group of variables were compared.

4. Permutation Analysis.

Method

The best AUROC was chosen from among these 127 (= 2^7^-1) variable combinations. However, it can be argued that by selecting the best of 127 patterns, we could have obtained favorable results purely by chance. Therefore, a permutation procedure was used to determine the probability of the observed results or more extreme results under the null hypothesis, by estimating the probability distribution of AUROCs of the best variable set under the null hypothesis. Because multiple samples obtained from patients were analyzed in this study, shuffling all samples as completely independent might have resulted in a biased estimation of the null hypothesis. Therefore, 1000 different disease label sets for 118 total samples (progressor plus non-progressor specimens) were generated randomly, while still accounting for correlated samples[10] (*i.e.,* multiple samples obtained from the same patient were labeled as identical). For each randomly assigned output label set (*i.e.,* each permutation), the best variable set was selected from among 127 (2^7^-1 for eight parameters) possible variable sets, using LOOCV. AUROCs of the best variable set from each permutation were recorded, and a distribution of these 1000 AUROCs represented the distribution of the null hypothesis. Next, descriptive statistics (mean, standard deviation, and maximum value) for the 1000 permutations were calculated, and histograms of the 1000 AUROCs were generated.

Result

**Figure S2** demonstrates the AUROC distribution of the null hypothesis, *i.e.,* the distribution of AUROCs that could have been obtained purely by chance (*viz.,* generated from randomly permuted labelings). The mean ± standard deviation and best AUROC value generated by random permutation were 0.6369 ± 0.0765 and 0.8602, respectively, for the 2-year model and 0.6191 ±0.0654 and 0.8148, respectively, for the 4-year model. The numbers of permuted AUROCs surpassing our original AUROCs (2-year model: 0.8387; 4-year model: 0.7910) were 3 and 4 for the 2- and 4-year models in 1000-fold permutations, respectively. Thus, the AUROCs of our original prediction model were significantly superior to the AUROCs of the null hypothesis, with false discovery rates (FDRs) of only 0.003 and 0.004 for the 2- and 4-year models, respectively. Therefore, this permutation procedure suggested that our observed results were unlikely to have occurred by chance.

Discussion

If a data analysis procedure includes the selection of best or better result(s) from a large number of data points, it risks selecting a good result that occurred by chance alone. For example, microarray analysis based on 10,000 genes will identify 500 as being significantly (p<0.05) differentially expressed, even if the data are completely random. To eliminate good results occurring by chance, one popular microarray analysis software, SAM[11], utilizes permutation analysis and estimates false discovery rates for each gene. In the current study, we chose the best parameter set based on 8 available molecular and clinical parameters. Then, we performed permutation analysis to validate our results by estimating the distribution of AUROCs that could have occurred by chance.

**References for Supplemental Materials**

1. Bureau USC (2006) Monthly Postcensal Resident Population, by single year of age, sex, race, and Hispanic origin. http://www.census.gov/popest/national/asrh/2005_nat_res.html

2. Ronkainen J, Aro P, Storskrubb T, Johansson SE, Lind T, et al. (2005) Prevalence of Barrett's esophagus in the general population: an endoscopic study. Gastroenterology 129: 1825-1831.

3. Dulai GS, Guha S, Kahn KL, Gornbein J, Weinstein WM (2002) Preoperative prevalence of Barrett's esophagus in esophageal adenocarcinoma: a systematic review. Gastroenterology 122: 26-33.

4. Reid BJ, Blount PL, Rabinovitch PS (2003) Biomarkers in Barrett's esophagus. Gastrointest Endosc Clin N Am 13: 369-397.

5. Spechler SJ (2003) The natural history of dysplasia and cancer in esophagitis and Barrett esophagus. J Clin Gastroenterol 36: S2-5; discussion S26-28.

6. Reid BJ, Levine DS, Longton G, Blount PL, Rabinovitch PS (2000) Predictors of progression to cancer in Barrett's esophagus: baseline histology and flow cytometry identify low- and high-risk patient subsets. Am J Gastroenterol 95: 1669-1676.

7. Korst RJ, Altorki NK (2003) High grade dysplasia: surveillance, mucosal ablation, or resection? World J Surg 27: 1030-1034.

8. Reynolds JC, Rahimi P, Hirschl D (2002) Barrett's esophagus: clinical characteristics. Gastroenterol Clin North Am 31: 441-460.

9. Inadomi JM, Sampliner R, Lagergren J, Lieberman D, Fendrick AM, et al. (2003) Screening and surveillance for Barrett esophagus in high-risk groups: a cost-utility analysis. Ann Intern Med 138: 176-186.

10. Braun T, Feng Z (2001) Optimal permutation tests for the analysis of group randomized trial. J Am Stat Assoc 96: 1424-1432.

11. Tusher VG, Tibshirani R, Chu G (2001) Significance analysis of microarrays applied to the ionizing radiation response. Proc Natl Acad Sci U S A 98: 5116-5121.
